# A Review of U-Net Based Deep Learning Frameworks for MRI-Based Brain Tumor Segmentation

**DOI:** 10.3390/diagnostics16040506

**Published:** 2026-02-07

**Authors:** Ayse Bastug Koc, Devrim Akgun

**Affiliations:** 1 Computer and Informatics Engineering Department, Institute of Natural Science and Technology, Sakarya University, Esentepe Campus, 54050 Serdivan, Sakarya, Türkiye; aysebastugkoc@uludag.edu.tr; 2 Department of Computer Programming, Gemlik Asım Kocabıyık Vocational School, Bursa Uludag University, Gemlik Sunğipek Asım Kocabıyık Campus, 16600 Gemlik, Bursa, Türkiye; 3 Department of Software Engineering, Faculty of Computer and Information Sciences, Sakarya University, Esentepe Campus, 54050 Serdivan, Sakarya, Türkiye

**Keywords:** brain tumor segmentation, BRATS dataset, U-Net, glioma subregions, U-Net variants

## Abstract

Automated segmentation of brain tumors from Magnetic Resonance Imaging (MRI) images is helpful for clinical diagnosis, surgical planning, and post-treatment monitoring. In recent years, the U-Net architecture has been observed as one of the most popular solutions among deep learning models. This article presents a review of 35 studies published between 2019 and 2025 focusing on U-Net-based brain tumor segmentation. The primary focus of this review is an in-depth analysis of commonly used U-Net architectures. The transformation of original 2D and 3D models into more advanced variants is examined in detail. Results from a wide range of studies are synthesized, and standard evaluation criteria are summarized along with benchmark datasets such as the BRATS competition to validate the effectiveness of these models. Additionally, the paper overviews the recent developments in the field, determines fundamental challenges, and provides insight into future directions, including improving model efficiency and generalization, combining multimodal data, and advancing clinical applications. This review serves as a guide for researchers to examine the impact of the U-Net architecture on brain tumor segmentation.

## 1. Introduction

Brain tumors are abnormal masses characterized by uncontrolled cell growth within or around the brain, leading to potential risks of morbidity and mortality [1]. They are life-threatening masses associated with high mortality and poor prognosis, requiring early diagnosis and treatment. Brain tumors can be primary, which originate in brain tissue, or metastatic, which spread from elsewhere in the body. Gliomas are the most common malignant primary brain tumors that begin in the glial cells of the brain. The World Health Organization classifies brain tumors into four grades based on their characteristics and growth [2]. Grades 1 and 2 are less malignant and slow-growing low-grade gliomas, while grades 3 and 4 are malignant and rapidly growing high-grade gliomas (glioblastoma multiforme). While benign tumors are not threatening, they can affect critical brain functions. Malignant tumors, also called cancers, are dangerous because they can spread. Therefore, early and accurate detection of brain tumors is an important step in treatment planning (surgery, radiotherapy, chemotherapy, response evaluation) and in predicting patient survival [3]. Magnetic Resonance Imaging (MRI) is the standard non-invasive method for analyzing brain tumors, as it does not use ionizing radiation and provides excellent soft-tissue contrast [4]. Clinical protocols typically use MRI sequences, including T1-weighted (T1), T1-weighted contrast-enhanced (T1-CE), T2-weighted (T2), and Fluid-Attenuated Inversion Recovery (FLAIR). Each sequence provides specific information where T1-CE highlights the active tumor core (enhancing tumor), while T2 and FLAIR are sensitive to peritumoral edema, with FLAIR suppressing signals from cerebrospinal fluid. Radiologists perform manual segmentation by carefully examining these modalities to outline different tumor sub-regions. However, this manual process is monotonous, time-consuming, and prone to variability between different observers [5]. Consequently, the development of fully automated segmentation methods has become an essential research area, primarily motivated by public benchmarks such as the Multimodal Brain Tumor Segmentation (BRATS) challenge [6]. Despite extensive efforts, accurate automated segmentation remains a difficult task due to the variations in tumor location, size, and shape, indistinct tumor boundaries, class imbalance, and variations in imaging protocols [7].

Fully automated brain tumor segmentation falls into two categories: traditional approaches and artificial intelligence-based approaches. Traditional techniques manually extract features (texture, shape, location, density, etc.) and feed them to machine learning algorithms. Due to the complexity of brain structure, these methods, which rely on human expertise, have failed to generalize. Recently, the rise of deep learning models that automatically learn features has increased their use in the medical field [8,9,10,11,12,13,14]. Deep learning models enable the extraction of local and global information from images and exceed manual feature engineering in segmenting complex tumor sub-regions.

U-Net is the most popular deep learning model for medical image segmentation [15,16,17]. The U-Net network is U-shaped and consists of two parts, the encoder and the decoder. The image size is reduced in the encoder part, and feature maps are generated using convolutional layers. The decoder increases the image size, and the feature maps are combined to produce the segmentation output. The decoder section reconstructs the boundaries of the object by restoring spatial information. There are skip connections with high-resolution details directly from the encoder to the decoder. These connections support segmentation by maintaining spatial details and preventing information loss. Many techniques used in brain tumor segmentation have been developed by modifying 2D and 3D U-Net architectures to improve performance. To enhance the U-Net’s segmentation performance, various structures have been incorporated into the original U-Net, including residual blocks, attention gates, dense layers, and transformer layers. 2D U-Net models are lighter and faster because they operate slice-by-slice on volumetric data, yielding slower but more accurate results. 3D U-Net models provide a more consistent context for tumor shape, location, and extent, thanks to volumetric convolutions, yielding more stable segmentation results than 2D models.

Brain Tumor Segmentation (BRATS) [5,6,18] competitions have played a crucial role in standardizing the segmentation of tumor subregions. The BRATS competition ensures comparability and standardization in the field of segmentation by using a common dataset, common preprocessing rules, and standard metrics (such as Dice score, Hausdorff 95 distance, Specificity and Sensitivity) for all research. The BRATS competition has enabled the development and benchmarking of automated algorithms specifically designed to address the challenges of gliomas and other brain tumors. In recent years, BRATS-winning models have improved segmentation performance by increasing accuracy through attentional mechanisms, deep supervision, cascading structures, and ensemble models that focus more effectively on the relevant region. This review examines 2D and 3D U-Net variation models for brain tumor segmentation on BRATS over the past six years (2019–2025), analyzing their methods, preprocessings, postprocessings, contributions, limitations, computational costs, performance results, and evidence appraisals. In addition, it discusses these studies and suggests new directions for future research. The remaining sections of the article are organized as follows: Section 2 describes the methodology for constructing the review. Section 3 presents the types of brain tumors and the segmentation methods used to segment them. Section 4 discusses commonly used and successful U-Net models. Section 5 summarizes the evaluation criteria, the BRATS dataset, and preprocessing and postprocessing for the BRATS dataset, and then describes selected studies on brain tumor segmentation using U-Net models. Section 6 discusses the current situation and identifies future trends. Section 7 presents the conclusions.

## 2. Review Method

This review thoroughly examines the studies that performed brain tumor segmentation on BRATS datasets, particularly using 2D and 3D U-Net architectures, between January 2019 and September 2025. This study presents a systematically conducted structured literature review using the Preferred Reporting Items for Systematic Reviews and Meta-Analyses Literature Search Extension (PRISMA-S) [19] reporting guidelines. It follows the PRISMA-S to ensure clarity in objectives, a detailed literature review, data presentation, and an evaluation of evidence. The study used explicit inclusion and exclusion criteria, searched multiple databases, and included a qualitative assessment to aid interpretation of the findings. The methodological framework is organized under three sections based on selected literature review principles: (a) research sources studied, (b) criteria for inclusion and exclusion, and (c) extraction and evaluation of pertinent information from the selected studies.

### 2.1. Research Source Study

Extensive searches were made in selected databases, including PubMed, Scopus, ScienceDirect (Elsevier), Frontiers, Google Scholar, IEEE Xplore, MDPI, SpringerLink, and Wiley Online Library. Only literature sources published within the last six years (January 2019–September 2025) were considered. Database-specific Boolean search strings were formulated by combining controlled vocabulary and free-text terms related to U-Net architectures, brain tumors, MRI imaging, and segmentation benchmarks. The primary search strategy was structured as follows:

(“U-Net” OR “UNet” OR “U Net”) AND (“brain tumor” OR glioma OR glioblastoma) AND (“MRI segmentation” OR “medical image segmentation”) AND (“BRATS” OR “BraTS dataset”).

Where necessary, this query was expanded using synonymous technical terms such as automatic delineation, automatic segmentation, semi-automatic segmentation, image processing, and deep learning to maximize retrieval sensitivity while maintaining relevance. All retrieved records were exported to a reference manager for duplicate removal prior to screening. Priority was given to high-quality studies characterized by meaningful experimental data and robust methodologies.

### 2.2. Inclusion and Exclusion Criteria

The BRATS dataset is an objective reference dataset for glioma segmentation, thanks to its multicenter nature, four different MR modalities, and clinically validated labels. The WT (whole tumor), TC (tumor core), and ET (enhancing tumor) regions, which represent different biological characteristics, are segmented separately, enabling better understanding of tumor structure and simplifying treatment planning. The dataset’s focus on adult gliomas may reduce the performance of developed models in pediatric cases or for different tumor types. However, the high variation in adult gliomas provides a basis for developing models in this field.

Using the same BRATS hypeparameters during both the training and testing phases of the models is important for ensuring consistent data distribution and reliable method comparisons. Dice scores are the most popular performance metric for evaluating tumor segmentation accuracy for its three subregions. Other metrics used in tumor segmentation include the Hausdorff distance, Sensitivity, and Specificity.

Studies relevant to this review were included based on the following criteria:

- The architecture utilized must be either 2D or 3D U-Net.
- The training and testing should have been conducted using BRATS, the most common benchmark dataset for adult glioma tumor segmentation.
- Segmentation of tumor subregions (WT, TC, ET) must be performed, and performance metrics (such as Dice, HD95, Sensitivity, Specificity) must be reported.
- Articles must have been published in peer-reviewed journals or international congress proceedings from January 2019 to September 2025 and must be in English. However, a few methodologically robust archive studies on BRATS datasets were also included.

Studies that met any of the following criteria were excluded from this review:

- Employment of other deep learning or machine learning techniques in brain image analysis.
- Experiments conducted on public or local datasets other than the BRATS dataset.
- Performance results for tumor subregions not reported.
- Publications not in English.
- Availability of the full text not confirmed.
- Repetitive content identified using different databases.

### 2.3. Data Extraction and Evaluation

**Figure 1:**
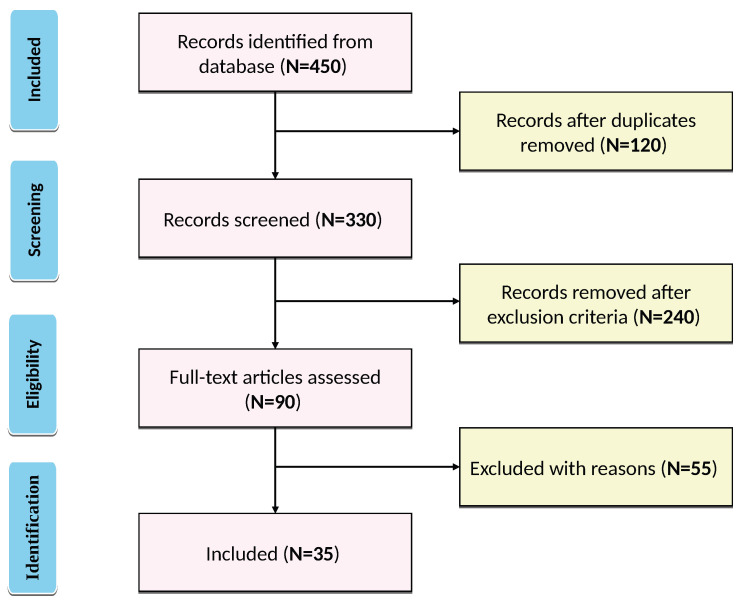
PRISMA-S diagram showing the literature review process.

Following a detailed literature review, duplicate entries were removed from all studies, titles and abstracts were examined, and a full-text scan was conducted to assess the suitability of the research for inclusion in this systematic review. Studies to be included and those to be excluded were systematically evaluated. The final selection yielded 35 research articles, as shown in the PRISMA-S flowchart in Figure 1. Important information was extracted from the selected studies and subsequently presented in both text and table formats in Section 5.4. The summary information derived from the studies contains the model descriptions with details on size and architectural modifications, the BRATS dataset utilized, the performance metrics results for tumor regions, computational costs, used preprocess and postprocess techniques, and the contributions, limitations.

## 3. Diagnosis and Segmentation of Brain Tumors

### 3.1. Brain Tumors and Their Types

Brain tumors are abnormal lumps in the brain where cells gather to multiply uncontrollably [20]. More than 120 types of brain tumors have been identified, but they are classified as primary and metastatic. Primary brain tumors are tumors that develop from brain tissue or nearby structures and account for more than 30% of all brain tumors. Primary tumors are categorized as benign or malignant based on their origin, whether glial (originating in brain cells) or non-glial (originating in the nerves, glands, and blood vessels within or on the brain structure). Tumors that develop in another organ, and typically spread to the brain through the bloodstream, are called secondary or metastatic brain tumors [21]. Malignant tumors that metastasize are considered cancer.

Gliomas (glial tumors) are one of the most common types of brain cancer in adults, accounting for almost 33% of all brain cancers (as much as 78% of malignant brain tumors). Glioblastoma is a difficult tumor for specialists to diagnose and treat. It is more common in men, typically between the ages of 50 and 70 [21].

Glioblastomas often have a mixture of cell grades and change as they grow. The characteristics of tumors seen under the microscope, along with their aggressiveness, allow specialists to identify tumor types. For example, lower grades indicate the least aggressive tumors, while higher grades indicate the most invasive tumors [22]. Brain tumor grades, types, and characteristics provided by the World Health Organization (WHO) [23]. In glioma, grades I and II are classified as Low-Grade Glioma (LGG), and grades III and IV as High-Gradeigh Grade Glioma (HGG).

According to the WHO report [24], the number of deaths from brain tumors in 2022 was 248,500, ranking 12th among cancer-related deaths. Additionally, according to the WHO report [24], the number of new cases of brain and central nervous system cancer in 2022 was reported as 321,731, and is expected to reach 443,000 in 2040, based on the WHO’s predictions for the future of cancer.

### 3.2. Brain Imaging Methods

Various imaging modalities such as Positron Emission Tomography (PET), Computed Tomography (CT), and Magnetic Resonance Imaging (MRI) are used for brain tumor diagnosis. PET visualizes metabolic activity using radioactive tracers, and CT provides cross-sectional X-ray images for detecting bone structures and acute bleeding. At the same time, MRI offers the most detailed representation of soft tissues and tumor boundaries [25]. Owing to its high soft-tissue contrast and lack of ionizing radiation, MRI is the preferred modality for brain tumor segmentation despite its higher cost and longer acquisition time [22]. MRI can be classified into functional MRI (fMRI), which measures brain activity through changes in blood flow, and structural MRI (sMRI), which captures anatomical and pathological features in axial, coronal, and sagittal planes [26]. This study concentrates on sMRI modalities, including T1, T1CE, T2, and FLAIR, each providing complementary tissue information. T1 images show normal anatomy, while T2 and FLAIR highlight water-rich areas such as edema and cerebrospinal fluid, with FLAIR concealing CSF signals to outline peritumoral edema better. T1CE improves visualization of active tumor regions by allowing gadolinium contrast to accumulate in areas of blood–tumor barrier disruption, enabling clear visualization of tumor margins and necrosis. The combined use of these modalities allows characterization of the tumor core and surrounding edema, improving segmentation accuracy [27].

### 3.3. Overview of Brain Tumor Segmentation

In computer vision, image segmentation involves dividing a digital image into multiple distinct parts, each with specific characteristics. The structure and boundaries of all objects are identified by assigning a particular class to each pixel in an image [28]. Segmentation can be divided into three subtasks: semantic, instance, and panoptic. In semantic segmentation, classification is performed at the pixel level, assigning each pixel a label. Instance segmentation detects each object of interest in an image, extracts its outline, and draws a bounding box around it. Panoptic segmentation, on the other hand, combines both semantic and instance segmentation to provide a better understanding. This method not only assigns a category label to each pixel, as in semantic segmentation, but also separately classifies different instances of objects belonging to the same class, as in instance segmentation [29].

**Figure 2:**
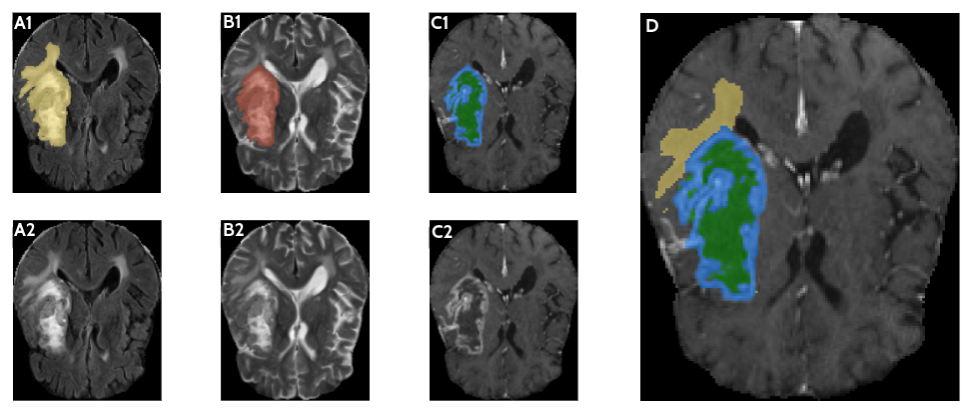
Tumor regions with MRI modalities ((**A1**) = WT overlaid on the FLAIR MRI slice, (**A2**) = FLAIR MRI slice, (**B1**) = TC overlaid on the T2 MRI slice, (**B2**) = T2 MRI slice, (**C1**) = ET overlaid on the T1CE MRI slice, (**C2**) = T1CE MRI slice) and their combination (**D**). From left to right: WT (yellow), TC (red), ET (blue), necrotic structure (green).

Medical image analysis typically involves heterogeneous data sampled from underlying anatomical and pathological processes. In the case of a glioblastoma brain tumor, the heterogeneous processes examined include the tumor itself, which consists of a necrotic (dead) portion and an active portion; edema or swelling in the surrounding brain tissue; and the brain tissue itself. This heterogeneity makes the analysis of glioblastoma challenging, as tissue transitions become unclear during the segmentation process and model stabilization is difficult. Not all glioblastoma tumors have a clear boundary between the necrotic and active regions, and some may lack a necrotic region altogether, further complicating matters. Multiple imaging modalities with varying contrasts are often used as a solution. Automatic segmentation methods aim to segment MR images into four types: normal tissue, WT, TC, and ET. WT includes peritumoral edema, ET, and necrosis; while TC includes necrotic and ET regions that constitute the tumor center. Identifying the ET region, the most active part of the tumor, is important in treatment and monitoring the tumor’s spread. Figure 2 illustrates the tumor regions using FLAIR, T2, and T1CE MR imaging modalities, and their combinations.

#### 3.3.1. Brain Tumor Segmentation Methods

**Figure 3:**
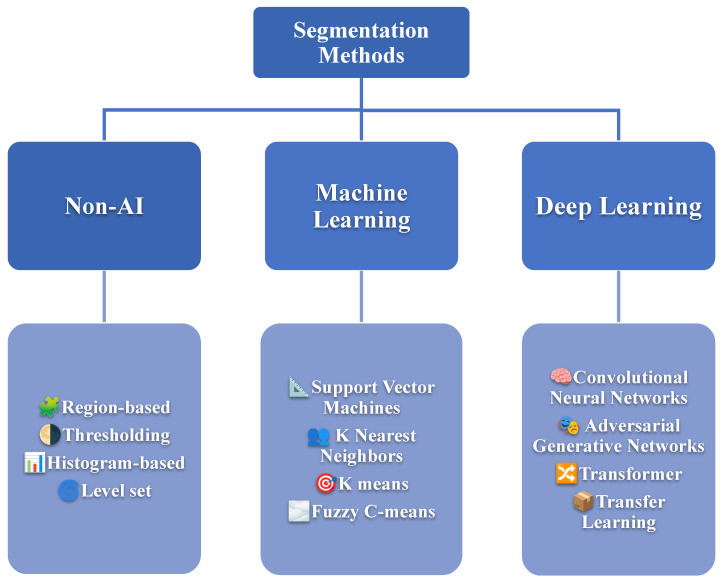
Approaches for brain tumor segmentation.

Brain tumor segmentation is classified into three categories based on the level of human involvement required: manual segmentation, semi-automated segmentation, and fully automated segmentation. While manual segmentation is important in both semi-automated and fully automated segmentation, it is labor-intensive and time-consuming. The accuracy of manual segmentation depends on the clinical expert’s training and experience [30]. Semi-automated segmentation combines computer and human expertise but requires human intervention. In fully automated brain tumor segmentation, artificial intelligence and prior knowledge are combined to solve segmentation problems [28]. The brain tumor segmentation methods can be divided into three groups: Non-AI, Machine Learning, and Deep Learning techniques, as illustrated in Figure 3. Notably, the number of studies based on deep learning for tumor segmentation exceeds other approaches.

Numerous non-AI methods, such as pixel-based, region-based, deformable models, histogram-based, and thresholding-based methods, have been used in medical image segmentation. These techniques have become popular due to their features, such as their applicability with small datasets, ease of use, low hardware requirements, and high domain knowledge [31]. Histogram-based models attempt to find a set of thresholds to distinguish objects and backgrounds [32].

Thresholding is an approach for converting color or grayscale images to binary images. While the method is simple, it can efficiently extract regions of interest from an image [33]. Manual intervention is required in the presence of noise and intensity variations, making it inefficient. [34]. However, this method struggles with objects with complex structures [27]. The region-growing technique is a simple, iterative, pixel-based image segmentation method that uses a predefined starting point, growth criterion, and stopping condition. This method is often used to detect tumor shape [35]. However, it has been reported that the method ignores half of the cerebral hemispheres and cannot address tumors in the midbrain [36]. Additionally, overestimating tumor volume is a common limitation of this approach.

Machine learning approaches involve different steps, including preprocessing, feature extraction, feature selection, and segmentation or classification. Supervised, unsupervised, and self-supervised learning are the primary types of machine learning. ML algorithms learn from labeled data in supervised learning, where each input data point has a corresponding output label. Unsupervised learning is when ML systems use unlabeled data to understand relationships among data. Standard features or patterns are sought in the data. Self-supervised learning is a machine learning technique that enables a model to learn from inherent relationships within the data. This learning method is based on the principle of hiding certain parts of the data and having the model predict this hidden information. ML has been used in neuroimaging to analyze brain cancers [37]. In the studies [38,39], a self-supervised learning approach was used to reduce the amount of labeled data and enable the model to learn more general feature representations. Support vector machines are supervised algorithms that find the optimal hyperplane with maximum margin for training samples. Researchers [40] have developed a method that improves generalization by using modified region-growing to expand the WT region and then extracting texture features. The best features are then selected and fed to the support vector machine for tumor type classification. However, the method can only perform WT segmentation and cannot handle simple binary or multi-class classification tasks. K-nearest neighbor attempts to find the closest match of a test vector in the feature space [41]. K-nearest neighbor performed less well than other machine learning methods in a brain tumor classification experiment [42]. Some drawbacks of the K-means algorithm have been identified, including random initialization of cluster centers, susceptibility to noise, increased computational complexity, inefficiency in complex tumor segmentation, and difficulty maintaining consistency for different MR images [43]. Fuzzy c-means is a clustering technique that assigns data points to multiple classes, yielding fuzzy features. However, many studies have reported its main challenges as being sensitive to noise, outliers, and complex tumor geometry [44]. For ML, features are extracted manually and fed into the ML system. The constant variation within image classes makes it difficult to use image classification algorithms. Furthermore, using modern distance metrics derived from feature extraction methods makes it impossible to determine the similarity between two images [37].

Deep learning is a form of machine learning and artificial intelligence that mimics how humans learn specific subjects. Many deep learning techniques have been developed and applied in brain tumor diagnosis in recent years. Among these, Convolutional Neural Network (CNN) is the most popular approach, and its use in brain cancer segmentation and classification has exceeded 70% [22]. In CNN, learning is done directly from the data, so there is no need to extract features manually. A CNN network can be trained using a large dataset, fine-tuning an existing model, or off-the-shelf CNN features [45]. CNN models can automatically discover hidden patterns in the input data and distribute weights among layers. However, these models may not be successful in detecting the orientation and location of objects [22].

Generative Adversarial Networks (GANs), proposed in the field in 2014, consist of a generator and a discriminator. The generator generates fake images, and the discriminator learns to distinguish them from real ones. With feedback from the discriminator, the generator learns to generate more realistic images [46]. It has been used to synthesize missing modalities and assist models in the field of brain tumor segmentation [47]. GAN models can improve data samples, increase data throughput, and improve models. A transformer is a typical attention-based deep learning model. Unlike locally connected CNNs, it can model long-range dependencies among markers, leading to better modeling of global feature relationships. Compared to CNNs, it typically has higher computational costs and has limitations, such as difficulty handling small dataset sizes, which can lead to overfitting [48]. Transfer learning has gained traction in medical image segmentation because it uses knowledge from pre-trained models on extensive natural image datasets. Transfer learning accelerates training and improves accuracy by integrating knowledge from large amounts of labeled data in relevant domains. However, there are also discussions regarding the transfer of learning from natural images to medical images [49]. The integration of deep learning into medical image analysis has the potential to change the roles of radiologists and clinicians, allowing them to spend less time analyzing medical images and more time on diagnostic and treatment decisions. Although deep learning techniques entail higher hardware and computational costs, they can yield remarkably accurate predictions when applied to standard datasets [31].

#### 3.3.2. Difficulties in Brain Tumor Segmentation

Volumetric brain MRI images are examined by specialists, including neurologists and radiologists, to segment brain tissue into various regions and identify tumors. This analysis is time-consuming and is not reproducible, primarily because it relies on the specialist’s knowledge and experience. Accurate segmentation of brain tumors is important for planning effective treatments, such as medication, surgery, and radiation therapy. Computer-aided analysis can help specialists identify tumors more quickly and produce consistent analysis results. Achieving correct analysis requires appropriate inputs for the computer-aided tools. However, several challenges arise during the segmentation process [25]:

- Due to radiofrequency emissions, raw MRI data may exhibit low signal-to-noise ratios and image artifacts. These fluctuations result in signal-dependent data drift and reduced image contrast [50].
- Non-uniformity, or additional and irrelevant intensity variation, can develop throughout the MR signal. Radio frequency coils can cause non-uniformity, the acquisition pulse sequence, and the geometry and structure of the sample.
- Along with brain images, there is unwanted information obtained by MR machines, such as skull, fat, and skin.
- The intensity profile of MR images may vary depending on the variety of MR machine configurations.
- Brain tumor images are rarely publicly available for computer-aided analysis. There are privacy or confidentiality issues when collecting MR images from various hospitals.
- Class imbalance is another important problem in medical image analysis. Finding images in abnormal classes can be difficult because they are rarer than normal classes.
- Manual labeling in the dataset depends on the individual expert’s experience, which may lead to annotation bias.
- Gliomas can arise anywhere in the brain due to the wide spatial distribution of the mutated, adherent cells from which they arise, making their location unclear. Furthermore, because brain tumor sites vary greatly in shape and size, predicting tumor morphology is difficult [51].

#### 3.3.3. Datasets Used in Brain Tumor Diagnosis

The distribution of datasets used by the studies is shown [27]. Most of the experiments are performed on the BRATS dataset (84%), followed by the local dataset (6%), Figshare (3%), and The Cancer Imaging Archive (TCIA) (3%).

The BRATS dataset [5,6,18] contains MRI images acquired using various imaging instruments and protocols that are based on tissue characteristics. Since its release in 2012, the BRATS dataset has been a widely used resource for brain tumor segmentation, providing a standard benchmark for evaluating and comparing segmentation algorithms. Annual data updates and competitions stimulate the design of accurate and reliable diagnostic tools for brain tumors.

Figshare is an online platform that enables researchers to share and discover their research outputs. It hosts various medical image sets for both medical research and computer-aided diagnostic algorithms. Among these resources is the brain tumor MRI dataset, which comprises 3064 T1CE images from 233 patients. This dataset includes 708 images of meningiomas, 1426 images of gliomas, and 930 images of pituitary tumors [52].

TCIA provides a complete repository for cancer-related medical images and related data, including datasets for brain tumors. The brain tumor collection includes datasets from 20 cases of primary newly diagnosed glioblastoma treated with surgery and standard concurrent chemo-radiation therapy followed by adjuvant chemotherapy. The purpose of this dataset is to evaluate the performance of a deep learning algorithm for predicting tumor progression [53].

The BITE database was developed to support the development and validation of new algorithms by sharing images of brain tumor patients. In 2010, preoperative and postoperative MRI and intraoperative ultrasound images obtained from 14 brain tumor patients at the Montreal Neurological Institute were added to the database. Each patient had a pre- and postoperative T1 MRI with gadolinium and multiple B-mode images before and after resection. Corresponding features were manually selected in some image pairs for validation. Experts assisted in selecting manual labels [54].

The Internet Brain Segmentation Repository (IBSR) is a dataset designed to simplify the evaluation and development of segmentation methods. It consists of manually annotated MRI images by experts. The task associated with this dataset is to segment brain tissues into gray matter, white matter, and cerebrospinal fluid.IBSR contains two T1 MRI images of healthy experimental participants: 20 (IBSR20) and 18 (IBSR18). Operators labeled both datasets to create the segmentation ground truth [52].

## 4. Widely Used Successful U-Net Models

**Figure 4:**
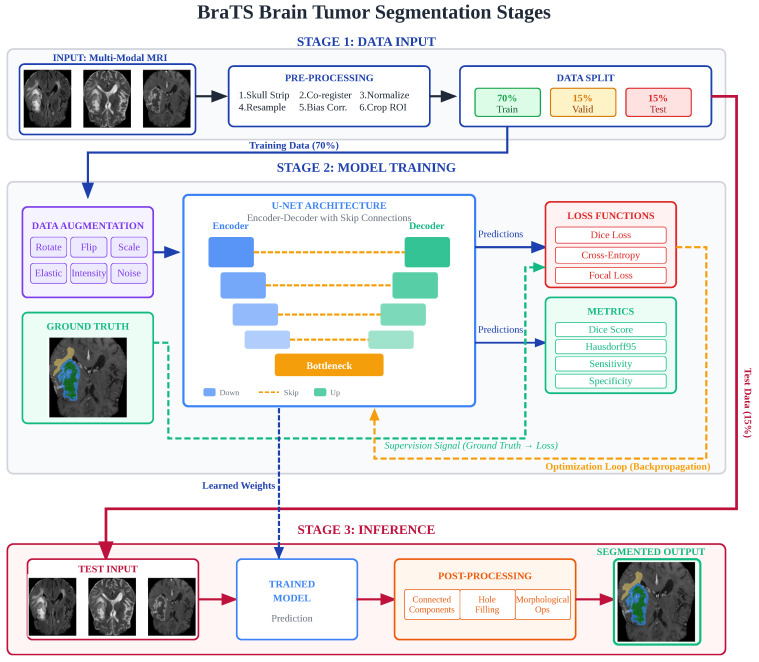
Illustration of brain tumor segmentation stages.

In deep learning, segmentation is a key process that divides an image into segments, each corresponding to a different object. Segmentation models analyze images to determine the boundaries of objects. The most common segmentation models used in deep learning are CNNs [55]. CNNs can learn spatial hierarchies of image features. In segmentation tasks, CNNs classify each pixel in an image and divide it into meaningful regions. Figure 4 illustrates a general segmentation process [56].

**Figure 5:**
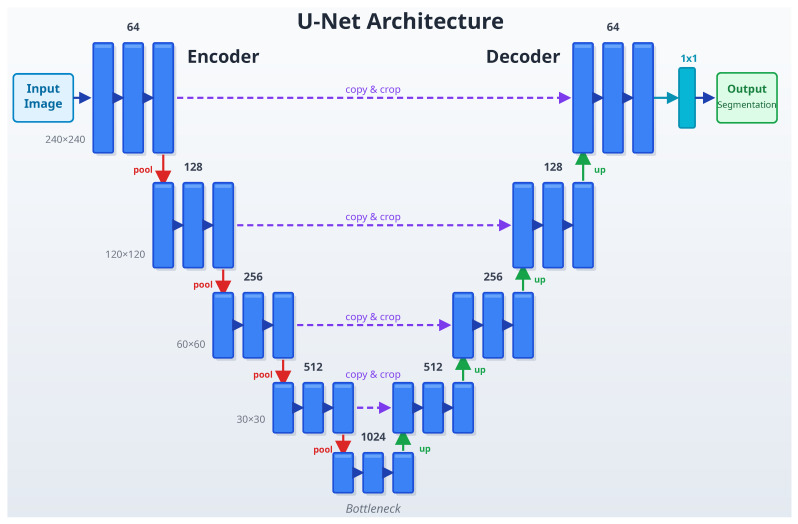
Typical U-Net structure.

Among CNN models, U-Net architectures, in particular, have revolutionized medical image segmentation. Ronneberger et al. created the U-Net [15] network in 2015, a model perfect for medical segmentation. U-Net was developed to break an image into smaller components, analyze these components, and then reassemble them, making it useful for detecting specific objects in medical images, such as organs or tumors. Because of the U-Net’s ability to simultaneously see the big picture and focus on small details, it is used by researchers in many fields for image analysis [57]. The 2D U-Net network typically consists of two symmetrical components: an encoder and a decoder, each with 4 or 5 levels. It is named after its U-shaped form due to its symmetric paths. The network architecture is shown in Figure 5 [15]. The encoder has two 3×3 convolutional layers that learn local texture features by expanding the receiver area at each level. Maximum pooling is applied after each sublevel. 2×2 Maximum pooling enables the extraction of deeper feature maps by halving the input resolution, providing a wider field of view. The goal of the encoding path is to extract the content of the input image for segmentation. At each level of the decoder, there are two 3×3 convolution layers arranged symmetrically with the encoder, which incrementally increase resolution through upsampling. A jumper connection is used to link the upsampling result to the output of the encoder submodule, which has the exact resolution as the input of the next submodule of the decoder. Finally, an additional 1×1 convolution operation is applied. The 1×1 convolution layer used reduces the large number of feature channels to the target class number and produces the final split image by assigning a class probability to each pixel.

Table 1 summarizes several architectural extensions, modifications, and advantages of the widely used U-Net variants.

## 5. Brain Tumor Segmentation with U-Net Models

### 5.1. Used Evaluation Metrics

The performance of a model is usually examined from many perspectives, such as accuracy, speed, and memory efficiency. However, most studies in the literature typically evaluate their models based on different accuracy metrics. This section provides a concise overview of commonly used segmentation accuracy metrics. While the presented quantitative accuracy metrics are an important tool for evaluating segmentation algorithms on various benchmark datasets, the visual quality of the model should not be overlooked, as the goal of these algorithms is to apply them to real-world problems [67].

- True Positive (TP): The pixel is correctly identified as a tumor region by the model.
- True Negative (TN): The pixel is correctly identified as healthy by the model.
- False Positive (FP): The pixel is mistakenly identified as a tumor region by the model.
- False Negative (FN): The pixel is mistakenly identified as healthy tissue by the model.

When evaluating segmentation models, various metrics are employed that provide unique information for accurately distinguishing between tumor and healthy regions [3]. Sensitivity, also known as the True Positive Rate or Recall rate, measures the percentage of actual tumor pixels correctly identified by the model. It aims to minimize missed diagnoses by ensuring the model detects all positive samples. Similarly, the True Negative Rate, also known as Specificity, measures the percentage of actual non-tumor pixels correctly identified by the model. These metrics are used to evaluate the accuracy of segmentation models in detecting tumor presence and absence. However, due to their sensitivity to segment size, their use as metrics for evaluating medical image segmentation is not widespread [68]. Sensitivity and Specificity are calculated using Equations (1) and (2).

(1) $$\mathrm{Sensitivity}=\frac{\mathrm{TP}}{\mathrm{TP}+\mathrm{FN}}$$

(2) $$\mathrm{Specificity}=\frac{\mathrm{TN}}{\mathrm{TN}+\mathrm{FP}}$$

Commonly used metrics for segmentation tasks are the Dice similarity coefficient or Dice score (Dice) and Hausdorff Distance [69]. The selection of the evaluation index can vary depending on the application needs. When considering boundary accuracy, the Hausdorff distance is a suitable option, whereas the Dice metric is better suited for overall segmentation accuracy and consistency. Model performance can be fully evaluated by considering various metrics. The two most important evaluation metrics for brain tumor segmentation are the Dice score and the Hausdorff distance [70].

The metric, also known as the Dice score or F1 score, measures the overlap between the predicted region (*B*) and the corresponding ground truth (*A*). Its value ranges from 0 (no overlap) to 1 (full overlap). This metric, widely used for its ability to handle class imbalance, is calculated using Equation (3) [3].

(3) $$\mathrm{Dice}\left(A,B\right)=\frac{2|A\cap B|}{\left|A|+|B\right|}$$

The Hausdorff distance (HD) measures the maximum distance between any point on the estimated segmentation boundary and the nearest point on the ground truth boundary. Its value ranges from 0 to infinity. The 95th percentile HD (HD95) is preferred to minimize the impact of outliers. The HD95 metric reflects the sensitivity of the model in detecting tumor boundaries and the accuracy of the boundary estimation. This metric is calculated using Equations (4)–(6). In these equations, *A* and *B* represent the ground truth and the prediction, respectively, while $A^{\prime }$ and $B^{\prime }$ denote the sets of voxel points in the two segmentation maps, and $a^{\prime }\in A^{\prime }$ and $b^{\prime }\in B^{\prime }$ represent individual voxel points [71].

(4) $$d\left(a,B^{\prime }\right)=\min_{b^{\prime }\in B^{\prime }}∥a-b^{\prime }∥,\;\forall a\in A^{\prime }$$

(5) $$d\left(b,A^{\prime }\right)=\min_{a^{\prime }\in A^{\prime }}∥b-a^{\prime }∥,\;\forall b\in B^{\prime }$$

(6) $$\mathrm{HD}95\left(A^{\prime },B^{\prime }\right)={\mathrm{percentile}}_{95}\left({\left\{d\left(a,B^{\prime }\right)\right\}}_{a\in A^{\prime }}\cup {\left\{d\left(b,A^{\prime }\right)\right\}}_{b\in B^{\prime }}\right)$$

### 5.2. BRATS Dataset

Since 2012, a multimodal brain tumor segmentation competition has been held annually in collaboration with the Medical Image Computing and Computer-Assisted Interventions (MICCAI). This competition evaluates the latest technologies in brain tumor segmentation. The dataset includes four different MRI modalities acquired from various institutions: T1, T1CE, T2, and FLAIR. All images were skull-removed, aligned, and manually labeled by expert radiologists. This established a publicly available standardized community metric for glioma segmentation. Between 2017 and 2020, the dataset was expanded to include overall survival predictions for patients. This enabled the models to perform radiographic analysis of glioma cases, thereby fulfilling a clinically valuable prediction task while also addressing the image processing problem. In 2021, the dataset was expanded to include the task of classifying the methylation status of the O6-Methylguanine-DNA Methyltransferase (MGMT) promoter in tumors. New clinical challenges were introduced with BRATS 2023. The dataset was expanded to include a broader case profile, including segmentation of underrepresented tumors from African regions and pediatric tumors; various tumor types, such as meningiomas and brain metastases; synthesis of missing MRI data; synthesis of healthy tissue; and evaluation of data augmentation. By including data from low- and middle-income countries, particularly Sub-Saharan Africa, the models’ global representativeness and generalization capabilities were tested on populations with different genetic backgrounds and varying device qualities. In BRATS 2024, the scope of research has been expanded to focus on pre- and post-treatment MRI images and their pathology to identify histological features that aid in diagnosing brain tumors, in addition to the BRATS 2023 tasks. Furthermore, significant improvements were made to the subsegmentation of classes to capture the nuances of tumor behavior for more efficient algorithm training. In BRATS 2025, the characterization scope has been expanded by adding more advanced tasks to the segmentation and synthesis tasks introduced in previous years, such as tumor response prediction, generalizability among different tumor types, prediction of microscopic differences in gliomas, and the inclusion of multicenter pediatric tumors. These tasks aim to go beyond static-image segmentation and integrate artificial intelligence into dynamic disease management and personalized medicine processes. Tumor subregions, previously divided into four classes: necrosis, edema, enhancing tumor, and non-enhancing tumor between 2012 and 2016, were reduced to three classes: necrosis, edema, and enhancing tumor in 2017. In BRATS 2023, BRATS 2024, and BRATS 2025, the dataset was diversified, with the addition of different annotation labels and tumor types, such as meningioma, metastasis, and pediatric, further complicating the tasks. This holistic approach aims to optimize healthcare delivery through personalized treatment plans, showing the potential of precision medicine [72].

BRATS is the most preferred data source by researchers in glioma detection and classification studies [73]. High-quality, annotated, and versatile MR images reflect the complexity of the real world and provide a valuable service to developing successful segmentation models. Table 2 briefly summarizes some features of adult glioma segmentation examples from the publicly available BRATS dataset, which is widely used for brain tumor segmentation.

### 5.3. Preprocessing and Postprocessing for BRATS

#### 5.3.1. Preprocessing

The BRATS datasets are distributed after standardized preprocessing, which includes co-registration to a standard anatomical template, resampling to an isotropic resolution of $1\;{\mathrm{mm}}^{3}$, and skull stripping. However, many workflows require additional preprocessing to reduce density variation between institutions’ scanning devices and improve robustness.

Cropping to the foreground: To reduce computational costs and address class imbalance in labeling, clipping is applied to non-zero voxels [74].

Intensity normalization: Crop intensities are adjusted according to percentile ranges (e.g., 0.5–99.5%, 1–99%), and min-max normalization or z-score normalization is applied to each MR modality of the participants using brain voxels. In the min-max normalization technique, the pixel intensities of MR images are normalized to the range [0, 1] to ensure consistent convergence during training. In the Equation (7), *X* represents the original pixel density value, $X_{n}$ the normalized value, $X_{\min}$ the minimum pixel density value, and $X_{\max}$ the maximum pixel density value [75].

(7) $$X_{n}=\frac{X-X_{\min}}{X_{\max}-X_{\min}}$$

To standardize data and eliminate anisotropic effects, Z-score normalization is applied, which involves subtracting the mean from each voxel and dividing by the standard deviation. This technique reduces overfitting and the occurrence of anomalous data, thereby accelerating convergence. The Equation (8) gives the formula for Z-score normalization [76]. The output and original input images are shown as $X^{\prime }$ and *X*, respectively. The average intensity level of the input image is shown as μ, while the standard deviation is shown as σ.

(8) $$X^{\prime }=\frac{X-\mu }{\sigma }$$

Bias-field correction: Artifacts frequently occur in MR images due to the uneven distribution of magnetic fields, variations in instrument sensitivity, and interactions between magnetic fields and the human body. These artifacts manifest as uniform variations in signal intensity within tissues with similar physical properties, thus compromising accurate image processing. SimpleITK or N4 Bias field correction can be used to correct this field aberration problem in the data [77].

Data augmentation: Data enhancement plays an important role in reducing the risk of overlearning and improving model generalization on the BRATS dataset, which has limited class-labeled data. Methods used in the literature for this purpose include affine transformations (translation, rotation, flipping, scaling, clipping, shearing), elastic/diffeomorphic deformations, pixel-level density transformations (Gaussian noise addition, brightness/contrast change, gamma correction), and artificial data generation Generative Adversarial Network (GAN) based approaches). Specifically, most BRATS participants prefer simple but effective affine and pixel-level enhancements. However, it is also emphasized that excessive or anatomically unrealistic enhancements can corrupt contextual information and negatively impact performance. Overall, the results from BRATS demonstrate that well-designed data enhancement strategies are a decisive factor in the success of deep learning-based brain tumor segmentation, regardless of the model architecture [78].

#### 5.3.2. Postprocessing

Postprocessing is commonly used to enforce anatomical plausibility, reduce false positives, and align outputs with BRATS evaluation conventions.

Ensembling and Test-time augmentation: Ensemble learning is employed to further improve segmentation performance by combining predictions from multiple independently trained models or model configurations. This strategy reduces model-specific biases and variance, resulting in more stable segmentation outcomes, particularly in heterogeneous tumor regions. Test-time augmentation is a practical approach to improving a model’s accuracy during inference. In this method, after training is complete, the test data is presented to the model in multiple variations by applying geometric and density-based transformations, such as rotation, mirroring, scaling, or adding mild noise. The multiple predictions are combined to produce the final segmentation map, yielding more consistent results, especially in ambiguous regions such as tumor boundaries. BRATS-based studies have shown that this process not only increases the Dice score but also enables evaluation of model uncertainty via inter-prediction variation [79].

Connected-component filtering (False-positive reduction): Reducing false positives in brain tumor image analysis is an important postprocessing step that enhances the reliability of the model’s results. Sometimes, computer models can incorrectly classify healthy tissue or noise in an image as a tumor. This technique eliminates these unrelated tumor-related areas by applying specific thresholding methods to the model-generated masks. This enables a more precise definition of tumor boundaries, thereby minimizing the margin of error, particularly in surgical planning and treatment [80].

Morphological cleanup and hole filling: Operations such as binary opening/closing, hole filling, and small-object removal can improve boundaries and remove pseudo-islands. These operations, classic tools in mathematical morphology, are typically adjusted on a validation set to avoid over-smoothing. Morphological operations are applied to ensure local consistency of segmentation and fill empty holes in the foreground (tumor) region [81].

Conditional Random Field (CRF): CRF is an effective technique for improving model output, particularly in structured prediction tasks such as medical image segmentation. Its primary goal is to produce smoother, more anatomically consistent results by considering not only the characteristics of a pixel or voxel but also its relationships with neighboring pixels and its spatial consistency when predicting the class of that point. In BRATS-based brain tumor segmentation studies, it has been demonstrated to reduce noise and improve boundary accuracy, particularly in regions with ambiguous tumor boundaries [82].

### 5.4. Selected U-Net Studies with the BRATS Dataset

This subsection presents selected recent studies that aim to improve accuracy by adding structures such as attention mechanisms, residual blocks, dense layers, and transformer layers to the U-Net architecture, along with their model configurations and parameter settings. Table 3 summarizes the current state-of-the-art models, preprocessing and postprocessing techniques for BRATS, and the computation cost of models. Table 4 summarizes the BRATS dataset and the performance metrics of models. The studies cover a variety of methodologies, including 2D/3D U-Net architectures, preprocessing enhancements, hyperparameter optimizations, and ensemble models. To enhance the interpretability of the literature reviewed and align it with PRISMA-S, a rigorous evaluation of the evidence for each included study is presented in Table 5. Each study was evaluated using four indicators: presence of ablation analysis, availability of publicly available code, external validation beyond the BRATS training set, and reporting of statistical significance testing. Based on these criteria, studies were qualitatively divided into three levels of evidence: preliminary (meeting 0 or 1 indicator), moderate (meeting 2 indicators), and strong (meeting 3 or 4 indicators). This classification aims to differentiate experimental architecture proposals from more precisely validated frameworks and to guide the interpretation of reported performance improvements.

Zhang et al. [83] developed a new architecture by adding attention gates and residual blocks to a 2D U-Net-based structure. Thanks to residual blocks and attention gates, high- and low-level features are better learned, while the class imbalance problem is avoided by a combination of Generalized Dice Loss (GDL) and Weighted Cross Entropy (WCE) loss functions. They stated that they aim to conduct future work on 3D MR data because 2D architectures cannot fully utilize the contextual information in 3D MR data. Punn and Agarwal [82] proposed a multimodality fusion 3D Inception U-Net model for segmenting brain tumors. MRI modalities were combined with inception blocks, features were extracted with the tumor extractor, and tumor subregions were segmented with the tumor segmenter. Using the Dice + Intersection-over-Union (IoU or Jaccard) loss function to reduce class imbalance during training improved accuracy but slowed convergence. Although the 10.5-million-parameter model provides robust results, it has a high computational cost. Yang et al. [80] proposed an improved 2D U-Net model with residual blocks. They extracted deep features using stepwise convolutions instead of residual blocks and pooling layers in the encoder/decoder section. They reduced overfitting with simple data augmentation (random rotation, translation, scaling, and cropping) and by adding a noise layer to the input, increasing the model’s diversity. They also used GDL and WCE loss to address the data imbalance problem. However, due to GPU memory capacity limitations, the compute size was kept small, and the 3D model could not be tested. Validation on larger, real-world clinical datasets is necessary.

Ullah et al. [84] examined different preprocessing techniques for brain tumor segmentation using the 3D U-Net architecture. They reported that the combination of bias field correction and Gibbs ringing artifact removal, which was used for the first time in the literature, increased Dice scores. However, they noted that the method performed poorly in tumor subregions, especially in the ET region, and suffered from data generalization deficiencies. Compared to existing techniques, they achieved similar performance in the WT and TC regions but were very low in the ET region. Feng et al. [85] developed an ensemble learning approach consisting of six general 3D U-Net models with experimentally selected different hyperparameters. To avoid errors arising from a single model, they combined the ensemble model with a multivariate linear regression model. They showed that the ensemble learning results were higher than those of single models. They expressed concerns about overfitting due to the dataset’s limited size. Wang et al. [86] proposed a 3D U-Net-based RDAU-Net with Convolutional Block Attention Module (CBAM) modules added to residual blocks, dilated convolutions (DA), and post-skip connections. They increased segmentation success by using residual blocks that avoid gradient loss and enhance feature extraction, dilated convolutions that provide different-scale feature maps and better capture tumor regions, and CBAM modules that reduce noise. They achieved higher Dice scores than existing successful methods, especially in the TC and ET regions. However, the performance improvement was limited in the WT region, and the added modules (DA and CBAM) introduced high computational costs. Guan et al. [87] developed a lightweight model that extracts multi-view spatial information using axial–coronal–sagittal fusion convolution and more accurately distinguishes complex boundary structures by representing lesions of different sizes with consistent parameters using kernel-sharing dilated convolution. Although the model demonstrated a good performance and computational stability in experiments, its generalization ability was insufficient when tested on limited datasets. The relationship between MRI modalities has been studied to a limited extent and tested with relatively small data sets. Yang et al. [88] proposed a new framework that combines the strengths of 2D U-Net and Mamba architectures. This is the first study in the field of brain tumor segmentation to combine the Mamba and U-Net architectures. Their architecture comprises the Selective Dual State Space Model (SD-SSM) and Spatial and Channel Reconstruction Convolution (SD-Conv). The SD-SSM block effectively captures local and global image features through selective scanning and state-space modeling. In contrast, the SD-Conv block increases computational efficiency by reducing redundancy. They also developed a loss function that combines mean IoU, Dice, and Boundary, increasing the sharpness of tumor boundaries during training and improving segmentation across many aspects. They achieved superior performance compared to current Transformer and U-Net-based architectures.

Wang et al. [89] improved segmentation performance by training a 3D U-Net based model with brain-based normalization that reduces background effects and two-stage patching strategies to achieve more efficient training; these strategies are more effective than classical normalization. Maji et al. [90] proposed a guided decoder architecture by adding redundant blocks and attention gates to a 2D U-Net to better learn tumor features. They guided the learning process by supervising each decoding layer using a loss function that combined weighted dice and EC. They improved prediction in small regions using a combined weighted loss function. The success of the model largely depends on the data augmentation and preprocessing steps. Jiang et al. [91] developed a sequential two-stage 3D U-Net that segments tumor regions from coarse to fine. In the first stage of this more complex stepwise structure, they generated segmentation maps using rough estimation, and in the second stage, they performed detailed segmentation with two decoders. They increased segmentation accuracy with a two-stage U-Net structure and strengthened the generalization ability of the encoder by using a dual decoder structure. The model, which also improves memory efficiency with a gradient checkpoint technique, achieved high performance. In their study, Li et al. [92] developed a three-stage approach using 3D U-Net for the WT and TC regions and 3D U-Net++ for the more complex ET structure, rather than a single network with multiple outputs to enable more efficient learning. Training was performed by selecting the most suitable MRI modalities at the input of each subnet, reducing parameter load. Clinicians found the results significant. Due to insufficient hardware resources, the cropping of input images and the use of small batch sizes resulted in reduced accuracy. Byeon et al. [93] presented a two-stage 3D U-Net model called Intelligence Cascaded U-Net (ICU-Net). In the first stage, they performed a coarse segmentation. In the second stage, they used the Expectation–Maximization Attention (EMA) module and dynamic convolutions to jointly learn global and local features, resulting in finer segmentation. Wang et al. [94] developed a lightweight, computationally efficient model called LHC-Net, based on the 3D U-Net architecture. Instead of using standard convolutions, they incorporated Res2Net and Residual Hierarchical Convolution (RHC) structures. The Res2Net architecture supports multi-scale feature extraction, enabling the model to segment tumors of different sizes simultaneously. Meanwhile, RHC helps prevent feature loss in layers, resulting in a more balanced representation of features. However, false-positive and false-negative predictions were generated due to shadows in small ET regions and near the sulcus. Rutoh et al. [95] enhanced the U-Net architecture by introducing initial residual blocks, a guided attention mechanism, and dilated convolutional layers to capture both local and global context while focusing on important regions. They further demonstrated the model’s generalization ability through cross-validation. While adaptable to variations in tumor morphology, it requires a large number of parameters and training time, which can be inadequate in some small tumor regions.

Aboussaleh et al. [96] combined the 3D U-Net, V-Net and Transformer architectures in their work, dubbed 3DUV-NetR+. More comprehensive information is captured by combining features obtained from the encoder sections of the U-Net and V-Net architectures. Transformer blocks added to the decoder levels represent long-range relationships. Combining CNN and transformers achieved results comparable to those of existing models. They aim to develop an online platform that can assist experts in the future. Since the dataset classes were unbalanced, the model’s performance was limited in the underrepresented classes. Zhang et al. [97] proposed a hybrid approach that captures both local (CNN-based) and global (Transformer-based) contextual information via specialized modules. To this end, they incorporated DepthWiseFormer (DWFormer) blocks into the encoder section of the basic 3D U-Net structure, Efficient Spatial-Channel Attention (ESCA) modules into the bottleneck section, and Multi-Scale Feature Cross Attention (MSFCA) modules into the skip connections. Although segmentation results are pretty high in the WT region, its ability to capture edge information in the irregularly bounded ET and TC regions remains low. Research into specialized loss functions is needed. Chen et al. [98] proposed a new architecture called the Adaptive Cascade Transformer U-Net, which has two stages (from coarse to fine segmentation). In the first stage, the basic 3D U-Net performs coarse segmentation. In contrast, in the second stage, it captures local details with dynamic convolutions and global information with the Swin transformer, achieving fine segmentation. They stated that the method could also find potential applications in medical fields beyond brain tumors. While Transformer structures are more efficient on large datasets, performance may degrade on small datasets. They also require postprocessing methods. Behzadpour et al. [99] extended the 2D Residual U-Net framework with a channel attention mechanism and integration of Atrous Spatial Pyramid Pooling (ASPP). This is the first study to segment a glioma using channel attention mechanism, ASPP modules, and residual blocks, achieving multiscale, competitive results. They improved feature extraction and parameter efficiency by using the EfficientNetB0 transfer-learning model as the encoder. The channel attention mechanism enabled better focusing on tumor regions, while the ASPP module provided multiscale contextual information.

Aumente Maestro et al. [100] enriched the 3D U-Net architecture with additional skip connections and deep supervision mechanisms, and performed a study using sample normalization, Leaky ReLU activation, and a limited number of convolution kernels (24, 48, 96, 192), demonstrating comparable performance to low computational cost heavyweight models. They stated that their two-stage approach, which first classifies tumor type (HGG/LGG) and then performs segmentation, is more accurate, especially in HGG groups. Wen et al. [77] developed a multitasking 3D U-Net-based framework capable of simultaneously grading and segmenting gliomas, along with a weighted combined loss function that best balances the two tasks. By integrating asymmetric convolutions and novel dual-domain attention mechanisms into the encoder, they efficiently extracted spatial and semantic features. They then used these features to perform segmentation and tumor grading simultaneously using two decoder structures. They noted that, due to its complex components, this model is sensitive to data quality and may delay rapid diagnosis in such situations. Further refinement of the model is also needed to expand the scope of experimental validation and reduce computational costs.

Bukhari and Mohy-ud-Din [81] proposed a novel and flexible variation of the classical 3D U-Net architecture, extending it with a single encoder and three independent decoders, using customized loss functions for the hierarchical subregions (WT, TC, ET) of the tumor. Their simple model, lacking complex structures and requiring no merging, outperformed some cutting-edge models, achieving superior results, particularly for the ET region. They published their code publicly. Separate training outputs depend on the postprocessing steps required to convert them to a multi-class problem. Luu and Park [74] presented a novel approach by extending the underlying model nn-UNet with modifications that don’t overload GPU memory. They increased model capacity by adding more filters, integrated a larger asymmetric encoder, and incorporated a decoder with an axial attention module to better capture areas of interest. They demonstrated the benefits of these additions by performing in-depth comparisons and using batch normalization to support decision-making during training. Hatamizadeh et al. [101] proposed a novel architecture, Swin Transformer, integrating a hierarchical encoder and a CNN-based decoder into a 3D U-Net structure. Their method, which effectively learns multi-scale features and contextual information, is the first to demonstrate the potential of transformers for brain tumor segmentation. They achieved results comparable to or better than those of the most advanced models currently available. However, it requires powerful hardware due to parameter density. Jia and Shu [102] developed a hybrid architecture that captures local and global features by integrating a 3D U-Net architecture with ViT blocks. Their new design uses a dual-transformer block in the encoder and decoder, a 3D CBAM-based attention block in the encoder, and transformer layers in the jumper connections. Tian et al. [103] developed a model that effectively captures tumor region boundaries by integrating the axial attention mechanism into the 3D U-Net architecture. They also reduced gradient loss with deep supervision and addressed class imbalance using a loss function that combines dice and binary cross-entropy. They stated that the proposed model demonstrates higher accuracy than existing methods and could be helpful for clinical use. They trained their computationally intensive models with a small batch size. In their study, Duman et al. [104] determined the best model using a region-focused (multi-label, multi-class, two-class) segmentation strategy and normalization (z-score and Nyul histogram matching) techniques, then combined them with a weighted ensemble learning technique. They demonstrated that it outperformed U-Net and nn-UNet architectures on the BRATS 2021 and local datasets, with shorter training time, lower memory usage, and an improved design. In their study, Alwadee et al. [105] introduced a lightweight 3D U-Net-based architecture incorporating parallel convolutions and attention mechanisms. Thanks to these parallel convolutions and attention mechanisms, they achieved both high performance and a computational load approximately 59 times lower than that of the most advanced models currently available. They interpreted their models using explainable artificial intelligence techniques. They observed errors, particularly in the segmentation of necrosis and edema regions. Ahmad et al. [106] presented a novel lightweight method using Inception-style parallel convolution blocks to capture multi-scale features with different kernel sizes (1×1×1, 3×3×3, 5×5×5) at each level based on a 3D U-Net. They addressed the class imbalance issue by combining Dice and focal loss during training, thereby improving accuracy in hard-to-detect tumor subregions.

Maani et al. [107] segmented adult glioma and pediatric tumors using an ensemble learning approach consisting of SegResNet and MedNeXt architectures on two 3D U-Net-based encoder and decoder frameworks. They systematically examined the effects of improvements provided by deep supervision and postprocessing methods. They produced more balanced predictions by combining 5-fold cross-validation training with probabilistic weighted averaging. Compared to the adult glioma dataset, they achieved lower performance due to the small number of pediatric samples. Kharaji et al. [108] proposed an extended version of the nnU-Net architecture using residual and attention blocks to overcome the limitations of the segmentation task on adult glioma and pediatric HGG datasets. The standard nnU-Net architecture learns complex representations with residual blocks, and attention gates suppress irrelevant regions and focus on tumor regions. They improved tumor edge accuracy by using a boundary-based HD loss function during training. They found that it demonstrated superiority, particularly on the adult glioma dataset, compared with the Kruskal–Wallis test. Their training period is longer than that of successful, basic models like nnU-Net and DeepMedic. Ferreira et al. [109] developed a new ensemble learning approach by combining the nnU-Net and Swin UNETR models. They bridged each other’s knowledge gaps by combining convolutional and transformer architectures. To increase data diversity and dimensionality, they generated synthetic data using a registration-based and GAN-based model and incorporated it into the BRATS 2023 adult glioma dataset.

In their work, Chen et al. [110] enriched the 3D U-Net structure with the transformed residual bottleneck structure of the MobileNetV2 model and the transformer design of the ConvNeXt model. The context was expanded with large convolutions (5×5×5, 7×7×7), while overfitting was reduced by using the Gaussian Error Linear Unit (GELU) activation function, group normalization, and orthogonal regularization in the layers. The limitations of the study include increased removal time due to the use of large convolutions and difficulty in identifying irregular tumor regions. Xing et al. [111] proposed a local-global U-Net transformer model based on the Swin UNETR architecture. They integrated efficient, low-parameter blocks into the encoder and semantically-oriented masked attention blocks into the decoder to improve learning. They achieved high-performance results. However, the number of parameters and GFLOPs is higher than that of pure U-NET and transformer models, making it unsuitable for resource-constrained devices. Jain et al. [112] conducted a study combining nnU-Net-based models and dynamically adding synthetic tumor data generated using GliGAN during training. The dynamic data augmentation process facilitated learning of rare lesions and reduced storage requirements by avoiding the generation of synthetic data in advance. Their results earned them first place in the BRATS 2025 challenge. GANs were used after processing MR data for synthetic data augmentation; excluding preprocessed MR data may limit generalization capacity.

**Table 3:** Summary of U-Net derived models, processing strategies, and computational cost on BRATS datasets (M: Million, NR: Not Reported, NU: Not Used).

Model	Preprocessing	Postprocessing	Computational Cost
2D U-Net with attention gate and residual block (AGResU-Net) [83]	Patch crop (128×128), Z-score normalization, Gaussian regularization	NU	NR
A 3D Inception U-Net encoder and 2-stage (tumor extractor, tumor segmenter) decoder developed with multimodality fusion architecture [82]	Bias field correction, Crop ($144^{3}$), Z-score normalization	CRF has been tested, but it has not been used because it reduces performance.	10.5 M parameter
Improved residual block 2D U-Net (DM-DA-UNet) [80]	Bias field correction, Random sampling (128×128), Z-score normalization, Data augmentation (rotate, scale, flip)	False positive reduction, Artifact removal	NR
3D U-Net using different preprocessing techniques on MR images [84]	Gibbs artifact removal, Bias-field correction, Intensity normalization, Adaptive histogram equalization	NU	NR
Ensemble learning consisting of 6 3D U-Net models trained with different hyperparameters [85]	Patch mean–std normalize, Data augmentation (Left-right flip)	Using Sliding Window + Overlap (For a Smooth Border), Flip-test time augmentation	Total training duration ≈ 60 h
Enhanced lightweight 3D U-Net with multi-view and core sharing mechanisms (MVKS-Net) [87]	Z-score normalization, Random cropping $128^{3}$, Data augmentation (rotation, intensity offset)	NU	0.5 M parameters, 28.56 GFLOPs
3D U-Net with CBAM modules added to residual layers, skip connections, and DA later (RDAU-Net) [86]	Bias field correction, Crop ($128^{3}$), Z-score normalization	Rescaling the output to its original size (240×240×155)	NR
Combination of Mamba and 2D U-Net (MUNet) [88]	Patch partition, Linear embedding, Normalization	Resolution recovery with Decoder + Patch Expanding	7.27 M parameters, 140.97 GFLOPs
3D U-Net using brain region-focused normalization and two-stage patching strategy [89]	Bias field correction, Normalization, Min-max scaling, Bounding box, 2-stage patching $128^{3}$	Combining patches into a single volume, Rescaling the output to its original size (240×240×155)	NR
2D U-Net with guided decoder, attention gate, and residual block (ARU-GD) [90]	Slice selection (25 from the middle), Z-score normalization, Data augmentation (flip, shift, rotate, zoom, shear)	NU	NR
Two-stage 3D U-Net [91]	Z-score normalization, Data augmentation (scale, shift, crop, flip)	Relabeling of small volume ET regions as Necrosis	NR
Three-stage 3D U-Net and 3D U-Net++ [92]	Bias field correction, Removal of brain region using Otsu method, Resizing to $128^{3}$ or $96^{3}$ volumes	Application of WT and TC masks to the ET phase in a cascade flow, Creating the final 3D tumor mask by combining the WT, TC, and ET outputs	Training duration ≈ 10 h/model
Intelligence cascade 3D U-Net (ICU-Net) [93]	Z-score normalization, Data augmentation (rotation, intensity shift, crop, flip)	NU	NR
Lightweight hierarchical convolutional 3D U-Net (LHC-Net) [94]	Clipping 0.5–99.5%, Bias field correction, Cropping $128^{3}$, Z-score normalization	Sliding window inference, Patch fusion, Threshold (0.5), Label transform	1.65 M parameters, 35.58 GFLOPs
Guided Attention Inception Residual 3D U-Net (GAIR-U-Net) [95]	Z-score normalization, Resizing $128^{3}$	NU	27 M parameters, 72.68 GFLOPs, Training duration ≈ 27.63 h, Inference time 3.5 s
Transformer integrated 3D U-Net and V-Net hybrid structure (3DUV-NetR+) [96]	Cropping $128^{3}$, Z-score normalization	NU	11.7 M parameters, Training duration ≈ 4.6 h
3D U-Net developed with efficient transformer modules (ETUNET) [97]	Cropping $128^{3}$, Data augmentation (intensity scale, crop, flip)	NU	16.26 M parameters, 101.81 GFLOPs
Adaptive two-stage transformer 3D U-Net (ACTransU-Net) [98]	Z-score normalization, adding an additional masking channel, Data augmentation (scale, crop, flip, brightness, noise, blur)	Sliding Window inference, Test time augmentation, Voxel cropping, Probability thresholding	35.9 M parameters, 655.7 GFLOPs
2D U-Net with transfer learning and channel attention blocking [99]	Data augmentation (rotation, shift, zoom, flip, nearest-neighbor interpolation)	NU	NR
Data-driven 3D U-Net for brain tumor segmentation (BTS U-Net) [100]	Clipping 1–99%, Voxel normalization, Cropping/padding (160×224×160), Data augmentation (flip)	False positive removal, Producing binary masks with a sigmoid + 0.5 threshold	5 M parameters, 1410 GFLOPs, Training time 168 s per epoch
3D U-Net with dual-branch, asymmetric convolution, and dual-domain attention mechanism (GSG-UNet) [77]	Cropping $128^{3}$, Bias field correction, Z-score normalization, Data augmentation (flip, rotate, translate)	NU	NR
3D U-Net with a single encoder multiple decoder approach (E1D3 U-Net) [81]	Z-score normalization, Random patching $96^{3}$, Data augmentation (flip, affine transformation, elastic deformation, gamma correction)	Morphological closing, Cluster thresholding, Hierarchical consistency constraint	NR
Extended 3D nn-UNet with axial attention and group normalization [74]	Z-score normalization, Data augmentation (rotate, scale, elastic deformation, gamma, brightness)	If the ET voxel count is <200, ET is removed and converted to Necrosis	NR
3D U-Net with Swin transformer encoder and CNN decoder (Swin UNETR) [101]	Z-score normalization, Patch crop $128^{3}$, Data augmentation (flip, intensity shift, intensity scale)	Sliding-window inference (overlap = 0.7)	61.98 M parameters, 394.84 GFLOPs
3D U-Net combined with ViT blocks (BiTr-Unet) [102]	Random crop $128^{3}$, Data augmentation (intensity shift, intensity scale)	Small region removal, Test-time augmentation, Model ensemble via majority voting	NR
3D U-Net with axial attention mechanism for brain tumor segmentation (AABTS-Net) [103]	Z-score normalization, Dynamic data augmentation (rotation, scaling, elastic deformation, gamma adjustment, brightness enhancement)	NU	NR
2D U-Net with region-based ensemble learning (RFS + Net) [104]	Slice/patch extraction, 2.5D 3-channel method, Z-score & Nyul normalization, Data augmentation (rotation, flip)	Deleting small regions, Ensemble model outputs, Resampling 3D masks to original size (240×240×155)	NR
A lightweight 3D U-Net with parallel convolutional attention blocks (LATUP-Net) [105]	Min-max normalization, Cropping $128^{3}$	NU	3.07 M parameters, 15.79 GFLOPs, Inference time 0.168 s
A lightweight 3D U-Net with inception blocks (LIU-Net) [106]	Z-score normalization, Cropping $128^{3}$	NU	3.124 M parameters, 58.66 GFLOPs, Training duration ≈ 20 h, Inference time 3 s
3D U-Net with SegResNet and MedNeXt ensemble learning [107]	Foreground cropping, Density normalization	Thresholding, Connected component analysis, Filtering tumor objects, Test-time augmentation	NR
Extended 3D nn-UNet with residual blocks and attention gates [108]	Z-score normalization, Random crop $128^{3}$, Data augmentation (rotation, scaling, gamma, noise, mirroring)	Removal of small components (nnU-Net default)	Execution time (Cycle time including inference + other processes) 119 s
3D nnU-Net and Swin UNETR ensemble learning [109]	Z-score normalization	Ensemble model, Threshold (removing small tumors)	NR
Transformer-driven 3D U-Net (TDU-Net) [110]	Z-score normalization, Resampling $128^{3}$, Data augmentation (flip, crop, gaussian noise)	NU	4.02 M parameters, 169 GFLOPs, Inference time 1356 s
Local-to-global 3D U-Net transformer (LG UNETR) [111]	Cropping $128^{3}$, Data augmentation (scale, flip, shift)	NU	83.9 M parameters, 1150.9 GFLOPs
3D nnU-Net ensemble learning [112]	Z-score normalization, Patch-based training (128×160×112), On-the-fly GAN augmentation	Lesion thresholding, Resection Cavity reset in pre-treatment patients	NR

**Table 4:** Quantitative performance comparison of U-Net-based brain tumor segmentation models on the BRATS datasets (NR: Not Reported) *.

Model	Dataset	Dice Score (WT-TC-ET)	HD95 Score (WT-TC-ET)	Sensitivity Score (WT-TC-ET)	Specificity Score (WT-TC-ET)
[83]	BRATS 2017 BRATS 2018 BRATS 2019	0.88-0.78-0.74 0.87-0.80-0.77 0.87-0.77-0.70	6.87-9.31-3.74 5.62-8.36-3.57 NM	NR NR NR	NR NR NR
[82]	BRATS 2017 BRATS 2018	0.89-0.88-0.83 0.92-0.91-0.84	NR NR	NR NR	NR NR
[80]	BRATS 2017 BRATS 2018	0.90-0.80-0.73 0.90-0.89-0.72	NR 12.20-8.53-4.15	0.89-0.77-0.75 0.90-0.81-0.79	0.99-0.99-0.99 0.99-0.99-0.99
[84]	BRATS 2018	0.90-0.83-0.71	NR	NR	NR
[85]	BRATS 2018	0.91-0.83-0.79	3.78-6.52-3.97	NR	NR
[87]	BRATS 2018 BRATS 2020	0.90-0.83-0.79 0.89-0.83-0.78	3.95-7.63-2.31 7.62-10.04-24.58	NR NR	NR NR
[86]	BRATS 2018 BRATS 2019	0.90-0.90-0.84 0.90-0.90-0.83	5.02-6.18-2.12 6.86-6.00-2.99	0.91-0.86-0.88 0.91-0.90-0.87	0.99-0.99-0.99 0.98-0.99-0.99
[88]	BRATS 2018 BRATS 2020	0.90-0.81-0.81 0.91-0.82-0.83	6.24-6.15-4.38 3.75-6.43-2.42	NR NR	NR NR
[89]	BRATS 2019	0.89-0.80-0.73	5.67-7.35-5.99	0.89-0.82-0.76	0.99-0.99-0.99
[90]	BRATS 2019	0.91-0.87-0.80 (HGG) 0.92-0.84-0.83 (HGG+LGG)	NR	NR	NR
[91]	BRATS 2019	0.88-0.83-0.83	4.61-4.13-2.65	NR	NR
[92]	BRATS 2019	0.86-0.83-0.80	6.14-4.92-6.75	0.90-0.83-0.84	0.98-0.99-0.99
[93]	BRATS 2019 BRATS 2020	0.89-0.82-0.78 0.90-0.82-0.78	5.91-7.77-3.39 4.57-14.58-35.03	NR NR	NR NR
[94]	BRATS 2020	0.90-0.83-0.76	6.96-6.30-30.09	NR	NR
[95]	BRATS 2020	0.88-0.86-0.85	NR	0.95-0.95-0.93	0.98-0.98-0.97
[96]	BRATS 2020	0.91-0.82-0.81	4.90-6.00-3.80	NR	NR
[97]	BRATS 2020	0.90-0.87-0.80	5.40-6.28-4.68	NR	NR
[98]	BRATS 2020 BRATS 2021	0.90-0.84-0.79 0.93-0.91-0.88	5.40-6.14-20.90 6.71-5.77-9.45	NR NR	NR NR
[99]	BRATS 2020	0.90-0.85-0.76	9.43-3.54-5.87	NR	NR
[100]	BRATS 2020	0.90-0.87-0.81	NR	NR	NR
[77]	BRATS 2020	0.91-0.85-0.85	4.29-6.75-5.10	NR	NR
[81]	BRATS 2021	0.91-0.86-0.86	5.68-17.36-9.51	NR	NR
[74]	BRATS 2021	0.92-0.87-0.84	3.47-7.62-20.73	NR	NR
[101]	BRATS 2021	0.92-0.87-0.85	4.73-15.30-16.32	NR	NR
[102]	BRATS 2021	0.92-0.93-0.88	3.00-2.23-1.41	NR	NR
[103]	BRATS 2019 BRATS 2021	0.91-0.83-0.77 0.92-0.86-0.83	3.98-6.02-3.24 3.99-11.17-17.72	NR NR	NR NR
[104]	BRATS 2021	0.92-0.87-0.84	NR	NR	NR
[105]	BRATS 2020 BRATS 2021	0.88-0.83-0.73 0.90-0.89-0.83	3.19-4.24-3.97 3.03-2.44-3.06	NR NR	NR NR
[106]	BRATS 2021	0.90-0.90-0.86	NR	NR	NR
[107]	BRATS 2023	0.85-0.82-0.81	34.12-39.86-35.1	NR	NR
[108]	BRATS 2023	0.90-0.81-0.79	3.10-3.90-4.40	NR	NR
[109]	BRATS 2023	0.87-0.75-0.75	26.32-35.61-34.5	NR	NR
[110]	BRATS 2021 BRATS 2023	0.89-0.88-0.83 0.92-0.91-0.85	3.99-3.43-2.75 3.86-3.21-3.79	0.88-0.87-0.84 0.92-0.89-0.86	NR NR
[111]	BRATS 2023 BRATS 2024	0.92-0.88-0.86 0.88-0.78-0.80	NR NR	NR NR	NR NR
[112]	BRATS 2025	0.88-0.79-0.79	NR	NR	NR

^*^ Studies are grouped and ordered by the BRATS challenge edition to reduce heterogeneity-related bias. Several publications report results using multiple BRATS datasets; all corresponding outcomes are presented to ensure clarity. This grouping does not imply cumulative performance advantage or strict ranking among studies, as each BRATS edition differs in cohort composition, annotation protocols, and segmentation difficulty.

**Table 5:** Methodological evidence appraisal of included U-Net-based brain tumor segmentation studies.

Study	Ablation Analysis	Code Available	External Validation	Statistical Significance	Evidence Level
[83]	Yes	No	Yes	No	Moderate
[82]	No	Yes	No	No	Preliminary
[80]	Yes	No	Yes	No	Moderate
[84]	No	No	Yes	No	Preliminary
[85]	No	No	No	Yes	Preliminary
[87]	Yes	No	No	No	Preliminary
[86]	Yes	No	No	No	Preliminary
[88]	Yes	Yes	Yes	No	Strong
[89]	No	Yes	No	No	Preliminary
[90]	Yes	Yes	No	Yes	Strong
[91]	No	No	No	No	Preliminary
[92]	Yes	No	Yes	Yes	Strong
[93]	Yes	No	No	No	Preliminary
[94]	Yes	No	No	No	Preliminary
[95]	Yes	No	Yes	No	Moderate
[96]	Yes	No	No	No	Preliminary
[97]	Yes	Yes	Yes	No	Strong
[98]	Yes	No	No	No	Preliminary
[99]	No	Yes	Yes	No	Moderate
[100]	Yes	Yes	Yes	Yes	Strong
[77]	Yes	No	No	No	Preliminary
[81]	Yes	Yes	Yes	No	Strong
[74]	Yes	Yes	Yes	No	Strong
[101]	No	Yes	Yes	No	Moderate
[102]	No	Yes	Yes	No	Moderate
[103]	Yes	No	Yes	No	Moderate
[104]	Yes	No	Yes	No	Moderate
[105]	Yes	Yes	Yes	No	Strong
[106]	Yes	Yes	Yes	Yes	Strong
[107]	Yes	Yes	Yes	No	Strong
[108]	Yes	No	No	Yes	Moderate
[109]	No	No	Yes	No	Preliminary
[110]	Yes	No	Yes	No	Moderate
[111]	Yes	Yes	Yes	No	Strong
[112]	Yes	No	Yes	Yes	Strong

## 6. Discussion and Future Directions

Since 2012, the BRATS competitions have expanded their structure and developed new brain tumor segmentation models. Since the U-Net architecture for medical image analysis was proposed in 2015, the most widely used and successful models in competitions have been 2D/3D U-Net variations. While 2D U-Net architectures are more efficient than 3D architectures in terms of computational efficiency and speed, their performance has been lower because they cannot accurately capture the spatial context of 3D architectures. Therefore, as GPU resources allow, most of the work has been on 3D U-Net variations.

The most common variants of U-Net architectures include residual layers, attention gates, dense layers, transformer layers, cascaded architectures, and ensemble learning. While these structures increase model complexity, they provide some accuracy and stability in detecting tumor subregions. The BRATS dataset, which suffers from limited data and class imbalance, is prone to overfitting. Therefore, attempts are made to reduce prediction errors by preprocessing the data, data augmentation, combining loss functions, and postprocessing methods.

The success of U-Net-based models in brain tumor segmentation depends not only on the architectural design but also directly on the quality of the preprocessing and postprocessing strategies applied. Since MR images are obtained from different centers, issues such as density heterogeneity and noise can be encountered in the raw data, necessitating preprocessing. This process typically involves noise reduction, skull scraping, bias field correction to normalize density differences between different modalities, and normalization techniques. Furthermore, to prevent overfitting in limited, unstable datasets such as BRATS, data augmentation techniques are used during training to improve model performance. After the training and prediction phases, postprocessing techniques can be applied to produce the final masks. Sometimes, anatomically impossible small pixels can generate false positive areas. Morphological processing and maximum coherence analysis applied at this stage ensure the removal of these noisy small parts. Furthermore, in the BRATS dataset, deleting clusters below a certain volume threshold directly increases segmentation accuracy by minimizing errors in tiny ET regions. In this case, while preprocessing prepares the model for the data, the postprocessing stage contributes to the segmentation process by making the model outputs consistent with medical reality, thereby enhancing the reliability of the segmentation throughout the final stage.

While the BRATS dataset provides researchers with a high-quality benchmarking environment using a standard template, a specific standard is often impossible due to the diversity of real-world clinical conditions. Differences in devices, protocols, resolutions, and parameters can lead to discrepancies between real-world data and the standard datasets used for training. This limits the generalizability of models and their validation on real-world clinical data. Furthermore, while there are successful applications in the segmentation of adult glioma tumors, the performance of models in other tumor types (such as pediatric, meningioma, metastases, and rare subtypes) is relatively poor. This is due to the underrepresentation of some clinical scenarios. In this context, domain adaptation and transfer learning techniques are important for future studies and can help address generalization problems. Performing model robustness tests using quasi-supervised learning techniques and MRI datasets with intentionally added artifacts and omissions can also help address real-world challenges.

Developing lightweight, fast, and efficient models is essential for resource-constrained environments. The black-box nature of artificial neural networks can hinder a complete understanding of their inner workings, potentially undermining confidence among physicians and patients. They may hesitate to use this technology because they cannot see the evidence on which the artificial neural network results are based. Explainable artificial intelligence (XAI) methods facilitate the interpretation of model decisions at both visual and analytical levels. The use of XAI methods will support the decision-making processes of medical professionals and increase confidence by enabling the transparent interpretation of results. Informative training can help medical professionals understand the use of artificial intelligence in decision-making processes, facilitate clinical integration, and improve trust and awareness. User-friendly interfaces enable the integration of AI models into clinical workflows with minimal effort, eliminating technical barriers. Easy access and use, simplified visual outputs, automated reporting, transparent interpretation, interactive segmentation tools, and time efficiency enhance clinician-AI collaboration.

While BRATS-based U-Net variants have made significant progress in recent years, computational efficiency, generalization, interpretability, and security remain to be addressed. Future work will focus on lightweight and efficient architectures, adaptation to different clinical domains, clinical validation, explainable AI additions, diversifying and expanding datasets to include other imaging modalities and genetic/molecular/personal information, and federative learning approaches. In situations where inter-institutional data sharing is difficult due to data privacy concerns, federated learning enables model weights to be centralized while raw data remains at the hospitals. This method enhances the model generalization by providing access to larger and different datasets. These improvements will increase the statistical accuracy of the model and enable reliable, efficient, real-time, and patient-centered use of AI-based approaches for medical purposes. Sharing local datasets and source code from publicly available studies to facilitate the reproducibility of results will provide another opportunity to accelerate the development of studies.

The qualitative evidence assessment, consistent with PRISMA-S, presented in Table 5, shows that, despite rapidly evolving architectural innovations in U-Net based brain tumor segmentation, most published studies remain at the preliminary validation stage. Many reported performance improvements are based only on assessments on the BRATS dataset, without validation on external datasets, statistical comparisons, or reproducibility in open-source applications, which increases concerns about the generalizability of highly optimized models and demonstrates that architectural complexity alone does not guarantee clinically important segmentation performance.

## 7. Conclusions

This review provides a detailed analysis of U-Net-based architectures for brain tumor segmentation, focusing on studies published between 2019 and 2025 that used the BRATS benchmark dataset. The growth of U-Net from the original encoder-decoder structure to advanced variants has been summarized. Combining architectural patterns, such as residual blocks, attention mechanisms, dense connectivity, and, more recently, Transformer-hybrid modules, has improved segmentation performance on benchmark metrics such as the Dice score, HD95, Sensitivity, and Specificity. On the other hand, there is still a research gap in segmenting small and irregularly bounded sub-regions, such as the ET, and in ensuring model generalization across different clinical sites, scanners, and imaging protocols. The class imbalance in the BRATS dataset poses a limitation that researchers have sought to address with advanced loss functions and data augmentation techniques. Finally, the development of U-Net frameworks suggests that the next frontier in brain tumor segmentation lies not in marginal gains in Dice scores, but in the robust deployment of these models within clinical environments. The future direction of this field should shift from incremental improvements in benchmark scores to tackling the practical problems of clinical implementation. Transitioning toward lightweight, interpretable, and federally trained architectures will be important to translating deep learning innovations into improved patient outcomes. Additionally, integrating XAI to enhance interpretability and using federated learning to protect data privacy will help successfully transfer these segmentation tools from research environments into clinical workflows.

## Figures and Tables

**Table 1:** Summary of widely used U-Net variants for MRI-based brain tumor segmentation.

U-Net Variant	Core Architectural Modification	Main Advantages
Standard U-Net	Symmetric encoder–decoder with skip connections and multi-scale feature fusion [15]	Preserves spatial details; strong baseline for medical image segmentation
U-Net++	Dense nested skip connections with deep supervision between encoder and decoder levels [58,59]	Reduces the semantic gap; improves boundary precision and feature representation
Attention U-Net	Attention gates integrated into skip connections to suppress irrelevant features [60,61]	Focuses on tumor regions; reduces false positives; improves ET and TC segmentation
Residual U-Net	Residual blocks with identity skip connections enabling deeper architectures [62]	Stabilizes gradient flow; enhances deep feature learning and convergence
Dense U-Net	Dense connectivity among convolutional layers enabling feature reuse [63]	Strengthens gradient propagation; improves fine-grained segmentation accuracy
Ensemble U-Net	Cascaded or multi-model U-Net predictions combined during inference [59]	Improves robustness; reduces variance; consistently increases Dice scores
Transformer U-Net	Hybrid CNN–ViT structures incorporating self-attention mechanisms [64,65,66]	Captures long-range contextual dependencies; improves global consistency

**Table 2:** Summary of adult glioma segmentation examples from the BRATS dataset. NA: Not Available.

Dataset	Number of Training–Validation– Test Samples	Image Size	Measurement time
BRATS 2012	35–NA–15	160 × 216 × 176 176 × 176 × 216	Before surgery/treatment
BRATS 2013	35–NA–25	160 × 216 × 176 176 × 176 × 216	Before surgery/treatment
BRATS 2014	200–NA–28	160 × 216 × 176 176 × 176 × 216	Before surgery/treatment
BRATS 2015	200–NA–53	240 × 240 × 155	Before surgery/treatment
BRATS 2016	200–NA–191	240 × 240 × 155	Before surgery/treatment
BRATS 2017	285–46–146	240 × 240 × 155	Before surgery/treatment
BRATS 2018	285–66–191	240 × 240 × 155	Before surgery/treatment
BRATS 2019	335–125–166	240 × 240 × 155	Before surgery/treatment
BRATS 2020	369–125–166	240 × 240 × 155	Before surgery/treatment
BRATS 2021	1251–219–570	240 × 240 × 155	Before surgery/treatment
BRATS 2022	1251–219–815	240 × 240 × 155	Before surgery/treatment
BRATS 2023	1251–219–570	240 × 240 × 155	Before surgery/treatment
BRATS 2024	1540–220–440	240 × 240 × 155	Before and after surgery/treatment
BRATS 2025	1296–179–303	240 × 240 × 155	Before and after surgery/treatment

## Data Availability

No new data were created or analyzed in this study. Data sharing is not applicable to this article.

## Abbreviations

The following abbreviations are used in this manuscript:

ASPP	Atrous Spatial Pyramid Pooling
BITE	Brain Images of Tumors for Evaluation
BRATS	Brain Tumor Segmentation (Challenge)
BT	Brain Tumor
CNN	Convolutional Neural Network
CRF	Conditional Random Field
CPU	Central Processing Unit
CSF	Cerebrospinal Fluid
CT	Computed Tomography
DA	Dilated Convolutions
DL	Deep Learning
EMA	Expectation–Maximization Attention
ESCA	Efficient Spatial-Channel Attention
ET	Enhancing Tumor
ETUNET	Efficient Transformer U-Net
FL	Federated Learning
FLAIR	Fluid-Attenuated Inversion Recovery
fMR	Functional MR
FN	False Negative
FP	False Positive
GAIR-U-Net	Guided Attention Inception Residual 3D U-Net
GAN	Generative Adversarial Network
GDL	Generalized Dice Loss
GELU	Gaussian Error Linear Unit
GFLOPs	Giga Floating-Point Operations Per Seconds
GPU	Graphics Processing Unit
GSG-UNet	Grading and Segmenting U-Net
HD	Hausdorff Distance
HD95	95th Percentile Hausdorff Distance
HGG	High-Grade Glioma
IBSR	Internet Brain Segmentation Repository
IoU	Intersection-over-Union (Jaccard Index)
LGG	Low-Grade Glioma
MGMT	O6-Methylguanine-DNA Methyltransferase
MICCAI	Medical Image Computing and Computer-Assisted Interventions
ML	Machine Learning
MR	Magnetic Resonance
MRI	Magnetic Resonance Imaging
MSFCA	Multi-Scale Feature Cross Attention

MUNet	Mamba U-Net
NR	Not Reported
NU	Not Used
PET	Positron Emission Tomography
RHC	Residual Hierarchical Convolution
PRISMA-S	Referred Reporting Items for Systematic Reviews and Meta-Analyses Literature Search Extension
sMR	Structural MR
T1	T1-weighted
T1CE	T1-weighted Contrast-Enhanced
T2	T2-weighted
TC	Tumor Core
TCIA	The Cancer Imaging Archive
TN	True Negative
TP	True Positive
ViT	Vision Transformer
WCE	Weighted Cross Entropy
WHO	World Health Organization
WT	Whole Tumor
XAI	Explainable AI
